# Utility of the six-minute walk test in patients with idiopathic pulmonary fibrosis

**DOI:** 10.1186/s40248-018-0158-z

**Published:** 2018-12-13

**Authors:** Lisa H. Lancaster

**Affiliations:** 1 Department of Medicine, Vanderbilt University Medical Center, Nashville, TN USA; 2 Division of Allergy, Pulmonary & Critical Care Medicine, Vanderbilt University Medical Center, 1301 22nd Avenue S, Suite B-817 TVC, Nashville, TN 37232 USA

**Keywords:** Exercise test, Interstitial lung disease, Oxygen

## Abstract

The six-minute walk test (6MWT) is a simple test that is widely used to assess functional exercise capacity in patients with idiopathic pulmonary fibrosis (IPF). Patients with IPF have reduced exercise capacity due to a number of factors, such as impaired respiratory mechanics and circulatory problems. As a self-paced and usually submaximal exercise test, the 6MWT reflects the exercise level of everyday activities. Variables measured during the 6MWT, such as six-minute walk distance (6MWD) and desaturation, are strong predictors of mortality in patients with IPF. However, the results of a 6MWT are affected by numerous factors, including age, body size, comorbidities and the use of supplemental oxygen during the test, and these need to be borne in mind when interpreting the results of individual and serial tests. Clinical studies, including trials of potential therapies for IPF, have differed in the methodology used to implement the 6MWT, hindering the comparison of results across studies. In this review, I describe the utility of the 6MWT in patients with IPF and provide recommendations for standardisation of the test for use both in clinical practice and research. A brief video on how to set up and administer the 6MWT is available at http://www.usscicomms.com/respiratory/lancaster/6mwt/

## Background

The 6-min walk test (6MWT) is a practical and objective measure of functional exercise capacity [1, 2]. A simple field test developed in the 1960s that measured the distance covered over 15 min to determine the exercise capacity of healthy individuals [3] led to the development of a 12-min walk test to assess disability in patients with chronic bronchitis [4]. A 6MWT for patients with respiratory diseases was developed to be less time-consuming for the investigator and less exhausting for the patient, and performed well compared with the 12-min test [5]. The 6MWT is widely used to assess functional exercise capacity, i.e., to determine the overall cardiopulmonary reserve, of patients with cardiac and pulmonary diseases, including heart failure, pulmonary hypertension, chronic obstructive pulmonary disease (COPD) and idiopathic pulmonary fibrosis (IPF).

During the 6MWT, an individual is asked to walk as far as possible over 6 min on a hard flat surface. In an effort to standardise the protocol, in 2002, the American Thoracic Society (ATS) issued practical guidelines for performing the 6MWT in patients with cardiovascular or pulmonary diseases, including scripted instructions for the patient and standardised phrases of encouragement [1]. In 2014, a European Respiratory Society (ERS)/ATS technical standard for performing field walking tests including the 6MWT was published, which included the same scripted instructions [2]. The primary measurement in the 6MWT is the distance walked i.e.*,* the 6-min walk distance (6MWD). A 30 m (100 ft) walking course, i.e.*,* a hallway, is recommended [1, 2], as shorter distances require the individual to reverse direction more often. When compared with the standard hallway method, a significantly lower 6MWD was obtained when a treadmill was used [6] and the use of a treadmill is not recommended [1, 2]. The 2002 ATS guidelines recommended measuring heart rate and perception of dyspnoea (Borg scale [7]) at the beginning and end of the test, with the option to measure arterial oxygen saturation (SpO_2_) using pulse oximetry at the beginning and end of the test, but not constantly [1]. However, the 2014 ERS/ATS technical standard recommended the continuous measurement of SpO_2_ during the test [2].

IPF is a progressive and ultimately fatal interstitial lung disease (ILD) characterised by loss of lung function and increasing dyspnoea [8]. Exercise capacity is markedly reduced in patients with IPF [9, 10], particularly in patients with advanced disease. During exercise, patients with IPF generally show an abnormal gas exchange response characterised by a dramatic fall in arterial oxygen i.e., oxygen desaturation and a lack of improvement in the efficiency of alveolar ventilation [11]. In a study investigating the mechanisms of gas exchange impairment in patients with IPF, patients at rest had moderate ventilation/perfusion (VA/Q) mismatching, with 4% of blood flow perfusing poorly ventilated lung units and 2% of blood perfusing completely unventilated lung units. Together these represented 81% of the alveolar-arterial oxygen (A-a) gradient. The other 19% was due to diffusion limitation in oxygen gas exchange. During exercise, arterial oxygen fell and the A-a gradient increased, with no change in the VA/Q mismatch, while the contribution of diffusion limitation in oxygen gas exchange increased to 40% [11].

In this review, I describe the utility of the 6MWT in patients with IPF, focusing on data from individual studies that support its use and the need for a standardised test to enable comparison of data across studies.

### Attributes and limitations of the 6MWT in patients with IPF

A summary of the key attributes and limitations of the 6MWT in patients with IPF is presented in Table 1.

**Table 1:** Key attributes and limitations of the 6-min walk test in patients with IPF

Attributes	Limitations
• Easy and inexpensive to perform	• Results affected by multiple factors ○ Age/sex/weight/height ○ Comorbidities that affect ability to walk or desaturation ○ Use of supplemental oxygen ○ Specific instructions given to patient, which may be affected by language barriers ○ Training effects
• Reproducible	
• Replicates activities of daily living	
• Provides a functional measure of the patient’s overall cardiopulmonary reserve	
• 6MWD correlates with measures of lung function and HRQL	
• 6MWD, and particularly decline in 6MWD, are strong predictors of mortality	
	• Provides no insight into mechanism/s of exercise limitation
	• Unclear which parameter measured during 6MWT is of greatest prognostic value
	• Use of different methodologies across studies hinders comparison of results

*6MWD* 6-min walk distance, *6MWT* 6-min walk test, *HRQL* health-related quality of life

### Relevance to activities of daily living

The self-paced 6MWT is considered a submaximal exercise test, in contrast to the cardiopulmonary exercise test (CPET), in which patients achieve maximal exercise capacity [1]. As most activities of daily living are performed at submaximal levels of exertion, the 6MWD reflects the functional exercise level of everyday physical activities [1]. In a retrospective study of 50 patients with fibrotic idiopathic interstitial pneumonia, including 29 patients with IPF, 6MWD was an independent predictor of the level of physical activity in patients’ daily lives [10]. Peak oxygen uptake (peak VO_2_) in patients with IPF undertaking a 6MWT and CPET is similar and 6MWD strongly correlates with peak VO_2_ during CPET [12]. However, there is variability in responses to the 6MWT and CPET according to the degree of lung function impairment, with the 6MWT imposing a greater cardiorespiratory load in patients with lower diffusing capacity of the lung for carbon monoxide (DLco) % predicted. Indeed, in patients with severe lung function impairment, the 6MWT may be a measure of maximal, rather than submaximal, exercise capacity [12].

The 6MWT evaluates the global integrated response of all the bodily systems involved during exercise, including the pulmonary system, cardiovascular system, systemic and peripheral circulation, blood, neuromusculature and muscle metabolism [1]. It does not provide information regarding the function of specific systems or the mechanisms of exercise limitation. Reduced exercise capacity in patients with IPF is multifactorial in nature, with contributions from both respiratory mechanics (e.g.*,* reduced forced vital capacity [FVC]) and circulatory impairment (e.g.*,* peripheral vascular resistance) [13]. Peripheral muscle dysfunction has also been implicated in reducing exercise capacity in patients with IPF [14].

### Correlation with measures of function and quality of life

There is a large body of evidence to support 6MWD as a measure that is relevant to patients with IPF and that assesses features of the disease that are related to, but not the same as, other commonly used measures of the severity and impact of IPF. Criterion validity of 6MWD has been demonstrated in two studies based on large cohorts of patients with IPF enrolled in clinical trials [15, 16]. At baseline, a greater 6MWD was associated with higher FVC % predicted, higher DLco % predicted, better health-related quality of life (HRQL) assessed using the St George’s Respiratory Questionnaire (SGRQ), lower dyspnoea (assessed using the University of California San Diego Shortness of Breath Questionnaire [UCSD-SOBQ]) and lower resting A-a gradient. However, these relationships were generally weak. Correlations between change in 6MWD and changes in FVC % predicted, DLco % predicted, dyspnoea, HRQL and A-a gradient at week 48 were stronger than correlations between these parameters at baseline [15, 16].

In smaller studies, 6MWD has been shown to correlate with lung function parameters in patients with IPF, including forced expiratory volume in 1 s (FEV_1_) [17], forced expiratory flow between 25 and 75% FVC [17] and vital capacity (VC) [18], and to correlate negatively with Medical Research Council (MRC) dyspnoea grade [19, 20]. The lowest SpO_2_ during the 6MWT, and the difference between SpO_2_ at rest and lowest SpO_2_ during the 6MWT, also correlate with measures of lung function (VC, FVC, DLco) [18]. In a retrospective study, 6MWD at baseline correlated positively with the physical function, role-physical and vitality domains of the Short Form 36 (SF-36) measure of HRQL, while lowest SpO_2_ during the 6MWT correlated positively with the general health, social functioning and mental health domains. Change in 6MWD over a median of 14 months correlated positively with changes in the bodily pain, general health, vitality and mental health domains, while changes in the lowest SpO_2_ during 6MWT correlated positively with changes in the physical function and general health domains [21].

### Prognostic value

Several studies have shown that 6MWD and/or decline in 6MWD are strong independent predictors of mortality in patients with IPF [15, 18–26]. In 822 patients with IPF and mild or moderate impairment in lung function who participated in a clinical trial, a 6MWD of < 250 m versus > 350 m at baseline was associated with a 2.65-fold increased risk of mortality over the following year, while a decline in 6MWD > 50 m versus 25 m over 24 weeks was associated with a 4.27-fold increased risk of mortality over the following year [15]. In an adjusted multivariate analysis in 454 patients with IPF listed for lung transplantation, a 6MWD < 207 m versus ≥207 m was associated with a 5-fold increase in mortality at 6 months [23]. In a study undertaken to develop models of disease progression using data from 1113 patients with IPF enrolled in three clinical trials, no multivariate prognostic model accurately predicted disease progression; however, lower baseline 6MWD was significantly associated with the risk of disease progression and death over 48 weeks [25]. The minimal clinically important difference (MCID) for 6MWD in patients with IPF has been evaluated as just 22–45 m [15, 16, 27, 28].

Desaturation (SpO_2_ ≤ 88%) during or at the end of a 6MWT and change in SpO_2_ during a 6MWT have been found to be significant predictors of mortality in some but not all studies in patients with IPF [18, 29, 30]. In one study in 81 patients, the distance-saturation product, a composite index comprising 6MWD and lowest room air SpO_2_ during the 6MWT, predicted mortality more accurately than either component alone [31]. In this study, a distance-saturation product < 200 m% versus ≥200 m% was associated with a 6.5-fold increased risk of mortality over 1 year and an 18% shorter median survival [31]. A study including 57 patients with ILD, of whom 36 had IPF, found that values at the end of the test failed to identify desaturation in 20% of patients [32]. These data, in combination with improvements in pulse oximetry technology, led to a recommendation for continuous monitoring of SpO_2_ during 6MWT to obtain an accurate measure of exercise-induced desaturation in the 2014 ERS/ATS technical standard [2]. Other variables measured during the 6MWT, including heart rate decline < 13 beats in the 1 min following the 6MWT [33], an impaired chronotropic response (peak heart rate – resting heart rate < 20 beats per minute) during the 6MWT [34] and Borg score at the end of the 6MWT [26] have also been shown to predict mortality in patients with IPF. Further research is needed to determine which variable measured during the 6MWT is of the greatest prognostic value.

### Ease of administration

The 6MWT is a simple test to administer, requiring no advanced training for technicians [1]. In patients with IPF, within-subject reproducibility is good both for 6MWD and for desaturation (SpO_2_ ≤ 88%) at the end of the 6MWT [29]. In a clinical study in 822 patients with IPF, the 6MWD showed stability between tests at screening and baseline, which were separated by a mean interval of 24 days [15].

### Factors affecting 6MWT

#### Patient characteristics

Being older, female, shorter and heavier can reduce 6MWD [1]. Several reference equations, based on data from healthy individuals, are available to calculate % predicted 6MWD adjusting for an individual’s age, sex, height and weight [35–38]; however, none of these has been validated in patients with IPF.

#### Oxygen supplementation

Patients who require it should be provided with oxygen supplementation during a 6MWT [1, 2]. The use of supplemental oxygen can lead to large increases in 6MWD, as well as prevent desaturation and reduce dyspnoea during the test [39, 40]. Oxygen flow titration should be conducted on a per-patient basis to ensure patients do not desaturate with the device being used [41].

Oxygen supplementation should be delivered in the same way and at the same flow rate in serial 6MWTs [1, 2]. Any change in the provision of supplemental oxygen to a patient should be considered in the interpretation of changes in 6MWD, and 6MWD from different studies should not be compared if oxygen was supplemented differently.

#### Comorbidities

Interpretation of 6MWT results in patients with IPF should be placed in the context of their comorbidities. This is particularly important when assessing elderly patients [42]. Musculoskeletal disorders can reduce 6MWD, as can cardiovascular disease and other pulmonary diseases [1]. Pulmonary hypertension (PH) is a common comorbidity in patients with IPF [43]. In an analysis of data from a clinical trial in patients with IPF, patients with IPF and PH associated with pulmonary disease (*n* = 68) had significantly lower 6MWD, resting SpO_2_ and minimal SpO_2_ during the 6MWT than patients with no PH (*n* = 374) [44]. However, results from small retrospective studies have yielded conflicting results as to whether concomitant PH reduces 6MWD in patients with IPF [45, 46]. Emphysema has also been shown to reduce 6MWD in patients with IPF; indeed in one small study, the extent of emphysema on high-resolution computed tomography (HRCT) scan was a stronger predictor of 6MWD than the extent of IPF on HRCT [17].

#### Instructions and training effects

Scripted instructions for the 6MWT are provided in the 2002 ATS guidelines and 2014 ERS/ATS technical standard, including stating that the objective of the test is to walk as far as possible for 6 min [1, 2]. In a small single-centre study, replacing ‘as far as possible’ with ‘as fast as possible’ led to a 66.5 m improvement in 6MWD in patients with IPF [47]. This may be because patients feel that they have to ‘pace themselves’ when asked to walk as far as possible to a greater extent than they do when asked to walk as fast as possible [47].

Serial 6MWTs are susceptible to training effects. In a study conducted in 30 patients with severely reduced exercise tolerance due to chronic respiratory disease, there was a significant increase in mean 6MWD across three 6MWTs undertaken on day 1, but no differences between the third walk on day 1 and 6MWTs performed on days 2, 3 and 14 [39]. The 2002 ATS guidelines state that a practice 6MWT is not required, but should be considered, allowing ≥1 h between tests and recording the highest distance [1]. The 2014 ERS/ATS technical document states that there is compelling evidence to support a learning effect in 6MWT results and that this is clinically important when change over time is being measured; in these circumstances, the ERS/ATS recommend that the better of two 6MWTs be used [2].

### Clinical trials using 6MWT as an endpoint

The 6MWT has been used as an endpoint in several Phase II/III trials of investigational treatments for IPF [48–61]. These have differed in the methodology used to implement the 6MWT, hindering the comparison of results across trials. Three Phase II/III trials have used a 6MWT or 6-min exercise test as a primary endpoint: a Japanese trial of pirfenidone [48], the BUILD-1 trial of bosentan [52] and the STEP-IPF trial of sildenafil [50]. In the Japanese trial, the primary endpoint was the change in the lowest SpO_2_ during a 6-min steady-state exercise test at 1 year [48]. Patients walked on a treadmill with the speed adjusted to that at which the patient could comfortably complete the test with a lowest SpO_2_ > 90%; that speed was used for the patient’s subsequent tests. At 9 months, the change in the lowest SpO_2_ during a 6-min steady state exercise test was + 0.47% with pirfenidone and − 0.95% with placebo (*p* = 0.07) [48]. In the BUILD-1 trial of bosentan, the primary endpoint was change from baseline in 6MWD at 1 year. The 6MWT was carried out largely in accordance with the ATS 2002 guidelines, but was terminated if SpO_2_ fell to < 80%. The mean change from baseline in 6MWD was − 52 m with bosentan and − 34 m with placebo (*p* = 0.226) [52].

In the STEP-IPF trial conducted in patients with DLco < 35% predicted and 6MWD > 50 m, the primary endpoint was the proportion of patients with a ≥ 20% improvement in 6MWD at week 12 [50]. The 6MWT was performed according to a standardised protocol, but not the ATS 2002 guidelines. Patients with a resting SpO_2_ ≥ 88% during screening undertook the 6MWT breathing ambient air, while patients with resting SpO_2_ < 88% received supplemental oxygen titrated to a resting SpO_2_ ≥ 92%. All 6MWTs were performed using the same amount of oxygen used at screening. The proportion of patients with a ≥ 20% improvement in 6MWD at week 12 was 10% in the sildenafil group and 7% in the placebo group (*p* = 0.39). Based on a linear mixed model, 6MWD decreased by − 28.5 m and − 45.2 m in the sildenafil and placebo groups, respectively (*p* = 0.11) [50]. A *post-hoc *analysis suggested that in patients with right ventricular systolic dysfunction, sildenafil was associated with a lower decline in 6MWD versus placebo (difference of 99.3 m [95% CI: 22.3–176.2]; *p* = 0.01), suggesting that there is a subgroup of patients with IPF who may benefit from sildenafil [62]. A recent Phase IIIb trial of nintedanib versus placebo in patients with IPF (NCT01979952) showed that after 6 months of treatment, the mean (SE) change from baseline in 6MWD was + 5(11) m in the nintedanib group versus − 13(11) m in the placebo group [63]. The 6MWT continues to be used as a secondary endpoint in Phase II and III clinical trials in patients with IPF (e.g. NCT02503657, NCT02550873, NCT02951429).

In addition to trials of potential therapies, the 6MWT has been used to investigate the impact of pulmonary rehabilitation and exercise training in patients with ILDs [64, 65]. In a randomised trial involving 142 patients with ILD, an 8-week supervised exercise programme was associated with a significant increase in 6MWD (25 m; 95% CI 2, 47 m) compared to usual care, with greater benefits observed in patients with worse 6MWD or symptoms at baseline [64].

### Recommendations for use of 6MWT in patients with IPF

Based on lessons learned from the literature, I make the following recommendations for a standardised approach to the use of the 6MWT in patients with IPF. A practice walk should be utilised prior to walks used to collect data in clinical trials. The language used for direction should be standardised. Taking into consideration that an increase in the number of turns increases the level of exertion, shorter hallway lengths (such as 50 ft. instead of 100 ft) may allow for greater compliance with future guidelines, as a 100 ft. hallway is difficult to find in many practices. How oxygen is carried, pushed or pulled needs uniformity as the mode of propelling oxygen requires varying degrees of exertion. Pulse oximetry should be continuously or periodically monitored during the 6MWT, with continued monitoring at least 3 minutes into the recovery period. Due to changes in cardiac output and perfusion when a patient goes from exertion to rest, further dips in oxygenation can occur during recovery.

Since intermittent demand oxygen is of little utility for patients with IPF who require continuous flow rates higher than 2 l/min, and confident standardisation of oxygen supplementation is needed, continuous flow oxygen should be used during the 6MWT for patients who have resting saturations of ≤88% or are known to desaturate with exertion. If patients desaturate to ≤88% during the 6MWT, an oxygen titration walk should be performed to quantify the flow rate of oxygen needed to maintain ≥90% saturation with exertion. A saturation of 90% is a reasonable goal considering the degree of error of the pulse oximeter. When the 6MWT is used in research studies, the same flow rate should be used throughout the study to allow comparison of variables over time while using the same oxygen supplementation. Other cardiopulmonary, neurologic and musculoskeletal diseases should be noted due to their impact on walk distance and velocity.

A brief video on how to set up and administer the 6MWT is available at www.usscicomms.com/respiratory/lancaster/6mwt/.

### Future research

The 6MWT is a rich source of data on disease progression that has not yet been fully assessed in patients with IPF or other pulmonary diseases. Beyond the 6MWD and oxygen saturation nadir, other variables may be telling of parenchymal disease (for example, change in desaturation from baseline to nadir, time to recovery of saturation after walk, distance desaturation product, heart rate recovery). Velocity during the entire walk, or in the first half versus the second half of the walk, may also be a marker of disease progression, as patients tend to slow down as they pace themselves. Further investigation of how the variables measured during the 6MWT relate to FVC decline and the risk of mortality in patients with IPF require further assessment. Composite variables may ultimately prove to be the most telling.

## Conclusions

The 6MWT is a simple test that is widely used to assess functional exercise capacity in patients with IPF. The 6MWT is relevant to patients’ performance of activities of daily living and correlates with measures of lung function and HRQL. Variables measured during the 6MWT, such as 6MWD and desaturation, are strong predictors of mortality in patients with IPF. However, as a global measure of exercise capacity, the results of a 6MWT are affected by numerous factors, including age, body size and comorbidities, and these need to be taken into account in the interpretation of the results. The use of supplemental oxygen during a 6MWT can lead to large increases in 6MWD, as well as prevent desaturation during the test. Comparison of 6MWT results between studies that varied in the methodology used to implement the 6MWT, for example in the approach to the use of oxygen, is unhelpful. There is a clear need to standardise the use of the 6MWT in patients with IPF for both research and clinical purposes.

## Abbreviations

6MWD: 6-min walk distance
6MWT: 6-min walk test
A-a: Alveolar-arterial oxygen (gradient)
ATS: American Thoracic Society
COPD: Chronic obstructive pulmonary disease
CPET: Cardiopulmonary exercise test
DLco: Diffusing capacity of the lung for carbon monoxide
ERS: European Respiratory Society
FEV_1_: Forced expiratory volume in 1 s
FVC: Forced vital capacity
HRCT: High-resolution computed tomography
HRQL: Health-related quality of life
ILD: Interstitial lung disease
IPF: Idiopathic pulmonary fibrosis
MCID: Minimal clinically important difference
MRC: Medical Research Council
PH: Pulmonary hypertension
SF-36: Short Form 36
SGRQ: St George’s Respiratory Questionnaire
SpO_2_: Arterial oxygen saturation
USCD-SOBQ: University of California San Diego Shortness of Breath Questionnaire
VA/Q: Ventilation/perfusion
VC: Vital capacity
VO_2_: Oxygen uptake
